# Weak binding to the A2RE RNA rigidifies hnRNPA2 RRMs and reduces liquid–liquid phase separation and aggregation

**DOI:** 10.1093/nar/gkaa710

**Published:** 2020-09-01

**Authors:** Veronica H Ryan, Scott Watters, Joshua Amaya, Balabhadra Khatiwada, Vincenzo Venditti, Mandar T Naik, Nicolas L Fawzi

**Affiliations:** Neuroscience Graduate Program, Brown University, Providence, RI 02912, USA; Department of Molecular Pharmacology, Physiology, and Biotechnology, Brown University, Providence, RI 02912, USA; Department of Molecular Pharmacology, Physiology, and Biotechnology, Brown University, Providence, RI 02912, USA; Department of Chemistry, Iowa State University, Ames, IA, USA; Department of Chemistry, Iowa State University, Ames, IA, USA; Department of Molecular Pharmacology, Physiology, and Biotechnology, Brown University, Providence, RI 02912, USA; Department of Molecular Pharmacology, Physiology, and Biotechnology, Brown University, Providence, RI 02912, USA

## Abstract

hnRNPA2 is a major component of mRNA transport granules in oligodendrocytes and neurons. However, the structural details of how hnRNPA2 binds the A2 recognition element (A2RE) and if this sequence stimulates granule formation by enhancing phase separation of hnRNPA2 has not yet been studied. Using solution NMR and biophysical studies, we find that each of the two individual RRMs retain the domain structure observed in complex with RNA but are not rigidly confined (i.e. they move independently) in solution in the absence of RNA. hnRNPA2 RRMs bind the minimal rA2RE11 weakly but at least, and most likely, two hnRNPA2 molecules are able to simultaneously bind the longer 21mer myelin basic protein A2RE. Upon binding of the RNA, NMR chemical shift deviations are observed in both RRMs, suggesting both play a role in binding the A2RE11. Interestingly, addition of short A2RE RNAs or longer RNAs containing this sequence completely prevents *in vitro* phase separation of full-length hnRNPA2 and aggregation of the disease-associated mutants. These findings suggest that RRM interactions with specific recognition sequences alone do not account for nucleating granule formation, consistent with models where multivalent protein:RNA and protein:protein contacts form across many sites in granule proteins and long RNA transcripts.

## INTRODUCTION

Spatial and temporal control of mRNA translation is crucial for highly coordinated biological processes like development and synaptic plasticity. RNA-binding proteins play a major role in regulating translation (1). The RNA-binding protein heterogeneous nuclear ribonucleoprotein (hnRNP) A2 is a well-studied component of granules that move mRNA from the nucleus to sites of local translation (2). hnRNPA2-containing granules were first studied in oligodendrocytes, where hnRNPA2 binds myelin basic protein mRNA and transports it to the oligodendroglial processes for local translation (3). hnRNPA2 binds a *cis*-acting element in myelin basic protein mRNA called the A2 response element (A2RE) in the 3′ untranslated region (3–5). The myelin basic protein mRNA consists of a 21-nucleotide sequence encoding two overlapping tandem A2REs of 11 nucleotides each (5,6). Similar, but not identical A2REs that bind hnRNPA2 have been identified in other transcripts, including mRNAs encoding ARC, neurogranin, alpha calcium calmodulin-dependent protein kinase II (αCaMKII), tau, and brain derived neurotrophic factor (BDNF) (7–9), demonstrating that hnRNPA2 has the ability to bind a variety of sequences. These transcripts are transported in transport granules containing mRNA, hnRNPA2 and other proteins (2). While we know the identity of many of these granule components, the architecture and interactions holding the granules together are unclear. At the same time, the discovery that hnRNPA2 can undergo liquid–liquid phase separation (LLPS) (10) has provided a model for how granules are formed, yet it is not clear how the presence of RNA affects these interactions. Some evidence suggests that RNA nucleates stress granule formation, but it is not clear if this is true of transport granules (11–13).

The structural basis of hnRNPA2 binding the A2RE is unclear. Recent work on the highly related protein hnRNPA1 showed that both tandem RNA recognition motifs (RRM) are required to bind the non-A2RE-like intronic splicing silencer at exon 7 (ISS-N1) of survival of motor neuron mRNA (14). The second RRM binds the 5′ region of the RNA studied and the RNA wraps around the RRMs, which sit almost back-to-back with substantial interacting surface area (14). In contrast, recent crystal structures (5EN1, 5HO4) of hnRNPA2 RRMs binding 8 or 10 nucleotide RNA sequences found that two hnRNPA2 molecules each consisting of 2 RRMs were required to bind two RNA molecules (15). Wu *et al.* found that RRM1 bound the 5′ region of the RNA and the RNA was stretched across two molecules of hnRNPA2, binding one RRM from each molecule of hnRNPA2 (15). Despite these different RNA arrangements, crystal structures of the related protein hnRNPA1 have RRM1 and RRM2 arranged in a similar configurations (<2 Å RMSD) (16–18). Further, recent work on hnRNPA1 RRMs binding a microRNA indicated 1:1 stoichiometry in solution experiments but a 2:2 stoichiometry in the crystal structure (18). Given the high identity of hnRNPA2 and hnRNPA1, as well as similar 2:2 crystal structures of hnRNPA1 (18,19) with 1:1 solution structures of hnRNPA1 (14,18), it seems possible that the proposed two protein to two RNA molecule stoichiometry of the hnRNPA2 crystal structure is the result of crystal packing contacts. Additionally, the RNA sequences studied are reasonably different from the A2RE, both in length and sequence, so hnRNPA2 may interact with the A2RE differently than these sequences. Hence, it is important to understand how hnRNPA2 interacts with the A2RE in solution in order to understand hnRNPA2 function.

Additionally, a number of RNA-binding proteins are known to be involved in neurodegenerative disease, in particular amyotrophic lateral sclerosis and frontotemporal dementia (ALS/FTD) (20). One hypothesis to explain the enrichment of RNA-binding proteins in genes mutated in ALS or FTD relates to the ability of many RNA binding proteins to undergo liquid–liquid phase separation (LLPS), forming protein-dense assemblies that may convert to protein aggregates in disease (21). LLPS is a phenomenon where proteins and nucleic acids demix from the surrounding solution to form a liquid phase distinct from the surrounding cytoplasm/nucleoplasm (*in vivo*) or buffer (*in vitro*). hnRNPA2 is capable of LLPS *in vitro* (10), but the effect of RNA binding on hnRNPA2 LLPS is unknown. hnRNPA2 is mutated in multisystem proteinopathy (MSP), a disease with elements of ALS and FTD as well as muscle and bone degeneration (22) and Paget's disease of bone (23). These disease mutations (D290V and P298L, respectively) are located in the disordered domain C-terminal to RRMs and cause hnRNPA2 to aggregate *in vitro* (10), but the effect of RNA on this process is unknown. Here, we use solution NMR and biophysical assays to determine the structure of the hnRNPA2 RRMs, how they bind to the A2RE, and how A2RE-binding affects phase separation and aggregation of full length hnRNPA2. These studies add to the structural information on hnRNPA2 and demonstrate that weak binding to RNA-binding proteins can prevent LLPS.

## MATERIALS AND METHODS

### Constructs

The following constructs and general purification strategies were used for protein expression in BL21 Star (DE3) *Escherichia coli* cultures (Life Technologies):

- hnRNPA2 1–189, soluble histag purification (Addgene ID: 157864)
- hnRNPA2 RRM1 (1–93), soluble histag purification (Addgene ID: 157865)
- hnRNPA2 RRM2 (94–189), soluble histag purification (Addgene ID: 157866)
- hnRNPA2 LC (190–341), insoluble histag purification (Addgene ID: 98657)
- C-terminal maltose binding protein tagged hnRNPA2 FL WT, D290V and P298L, soluble histag purification (Addgene ID: 139109, 139110, 139111 respectively).

### Bacterial culture and isotope labeling

Uniformly ^15^N- or ^13^C-labeled proteins were expressed in M9 in H_2_O with ^15^N ammonium chloride as the sole nitrogen source or ^13^C-glucose as the sole carbon source as appropriate. Unlabeled proteins were expressed in LB. Cell pellets were harvested from 1 l cultures induced with IPTG at an OD600 of 0.6–1 after 4 h at 37°C. Pellets were resuspended in 20 mM NaPi pH 7.4, 1 M NaCl, 10 mM imidazole (∼30 ml per pellet from 1 liter culture) with a Roche Complete EDTA-free protease inhibitor tablet or a Pierce protease inhibitor tablet. Resuspended pellets were lysed on an Emulsiflex C3 and the cell lysate cleared by centrifugation (20 000 × g for 60 min at 4°C).

### Purification

Cleared RRM lysate was filtered and loaded onto a HisTrap 5 ml column. Protein was eluted in a gradient of 10–300 mM imidazole over five column volumes. Fractions containing hnRNPA2 RRM was pooled and buffer exchanged to reduce imidazole concentration to <50 mM. Protein was incubated with TEV protease overnight at room temperature. After TEV cleavage, protein was loaded onto a 5 ml HisTrap column and flow through containing cleaved hnRNPA2 RRM was pooled, concentrated, and loaded onto a Superdex 75 column equilibrated in 20 mM NaPi pH 7.4 1 M NaCl. Fractions containing undegraded protein were pooled, concentrated, and flash frozen. Protein was effectively free of nucleic acid contamination as *A*_260_/*A*_280_ UV absorbance ratios were 0.55–0.60.

Cleared full-length hnRNPA2-MBP lysate was filtered and loaded onto a HisTrap 5 ml column. Protein was eluted in a gradient of 10–300 mM imidazole over five column volumes. Fractions containing hnRNPA2-MBP were pooled and loaded onto a Superdex 200 equilibrated in 20 mM NaPi pH 7.4 1 M NaCl. Fractions containing full length protein without degradation products were pooled and protein was concentrated to ∼1 mM and flash frozen. Protein was effectively free of nucleic acid contamination as determined by *A*_260_/*A*_280_ ratios of 0.5–0.6.

hnRNPA2 LC was purified as described (10). Briefly, the insoluble pellet was resuspended in 8 M urea, cleared, filtered, and loaded onto a 5 ml HisTrap column. Protein was eluted in a gradient of 10–300 mM imidazole over five column volumes. Pooled fractions containing protein were buffer exchanged into 8 M urea pH 5.5 20 mM MES diluted with 20 mM MES pH 5.5 to a final urea concentration of 500–1000 mM, and incubated with TEV overnight. Cleaved protein was solubilized in 8 M urea, loaded onto the HisTrap column and flow through containing cleaved hnRNPA2 LC was pooled, concentrated, buffer exchanged into 8 M urea pH 5.5 20 mM MES and flash frozen.

### RNA and DNA

RNA was ordered from Horizon Discovery (formerly Dharmacon, https://horizondiscovery.com/products/tools/Order-Single-strand-RNA) with HPLC purification and 2′ deprotected and desalted by the company. The sequences are: rA2RE11 GCCAAGGAGCC, rA2RE21 GCCAAGGAGCCAGAGAGCAUG, rA2RE21^scr^ GGACGACAGGACGCGAGCUAA, Cy3-rA2RE11 Cy3- GCCAAGGAGCC, Wu_10mer AAGGACUAGC, long_rA2RE21 UGCGGAUAGACAGGCACACCGCCAAGGAGCCAGAGAGCAUGGCGCAGGGGACUGUGUGGU, and long_rA2RE21^scr^ UGCGGAUAGACAGGCACACCGGACGACAGGACGCGAGCUAAGCGCAGGGGACUGUGUGGU. DNA was ordered from Invitrogen.

### ITC

Isothermal titration calorimetry (ITC) experiments were performed on a VP-ITC instrument (MicroCal Inc/Malvern). Absorbance at 280 and 260 nm was used to determine the concentrations of proteins and RNAs, respectively. Sample concentrations were estimated using the extinction coefficients calculated by ProtParam. Samples were prepared in 20 mM NaPi pH 6.75, 150 mM NaCl; 20 mM KPi pH 6.75; or 20 mM MES pH 6.7, 150 mM NaCl, 10 mM CaCl_2_ as appropriate. Samples were degassed for 15 min prior to titration using a Thermovac (MicroCal Inc./Malvern). The measurements were performed using the protein as titrant (syringe) and the RNA as titrand (cell). All measurements were performed at a cell temperature of 25°C over at least 27 injections starting with at least one 2 μl initial injection (as indicated where additional 2 μl initial injections were added). All other injections were 10 μl. Spacing time was 240 s for most runs except the experiment in the CaCl_2_ buffer, where it was 300 s. The filter period was 2 s and stir speed was 307 rpm for all runs. The reference power was set to 30 μcal/s and the initial delay to 60 s. The data was analyzed using Origin version 7.0 (MicroCal Inc). For A2RE11 and the Wu_10mer, a binding model using nonlinear least-squares fitting and one binding site was applied.

### Phase separation/aggregation

Samples of full-length hnRNPA2-MBP were prepared for microscopy by diluting proteins from 1 M NaCl buffer to final protein and salt concentrations. Solid RNA was dissolved in the volume of 20 mM Tris pH 7.5 calculated to give 1 mM RNA concentration based on the RNA weight on the tube. Final RNA concentration was determined by absorbance at 260 nm using NanoDrop and the given extinction coefficient for the RNA oligomer. 50 μl samples of 15 μM protein in 20 mM Tris pH 7.5, with a final NaCl concentration of 50 or 150 mM as appropriate were prepared for each time point. TEV was added and allowed to cleave for indicated time. 20 μl of sample was spotted onto a coverslip and the water drop imaged. DIC images were taken with an Axiovert 200M microscope (Zeiss). Phase separation experiments with hnRNPA2 LC were performed as in (10) with the addition of fluorescently labeled RNA at given concentrations dissolved in pH 5.5 20 mM MES.

### NMR

#### Solution NMR samples

hnRNPA2 1–189 NMR samples were made by diluting protein from stocks containing 1 M NaCl into 20 mM NaPi pH 6.8 with 10% ^2^H_2_O to a final salt concentration of 150 mM. Sample concentrations were estimated using the extinction coefficients calculated by ProtParam.

#### Solution NMR experiments

NMR experiments were recorded at 298 or 310 K as indicated using a Bruker Avance NMR spectrometer operating at 850 MHz ^1^H frequency or 500 MHz ^1^H frequency as indicated. Experimental sweep widths, acquisition times, and the number of transients were optimized for the necessary resolution, experiment time and signal to noise for each experiment type.

#### hnRNPA2 1–189 Assignments

Triple resonance assignment experiments were performed on samples of ^13^C/^15^N uniformly labeled hnRNPA2 1–189 (conditions: 20 mM NaPi pH 6.8 150 mM NaCl with 10% ^2^H_2_O). CBCA(CO)NH, HNCACB, HNCA, HCNO and HN(CA)CO were recorded with sweep widths 16 ppm (center 4.7) in ^1^H, 34.5 ppm (center 116) in ^15^N, 14 ppm (center 176) in ^13^C for CO experiments and 61 ppm (center 43) in ^13^C for CA/CB experiments using standard Bruker Topspin3.5 pulse programs with default parameter sets (cbcaconhgp3d, hncacbgp3d, hncagp3d, hncacogp3d, hncogp3d). Experiments comprised 68, 64, 56 and 1536 points in the indirect ^15^N, indirect ^13^Cα/Cβ, indirect ^13^CO and direct ^1^H dimensions respectively.

#### NMR chemical shift deviations

^1^H–^15^N HSQCs of 200 hnRNPA2 1–189 with indicated ratios of RNA were acquired at 850 MHz ^1^H Larmor frequency or 500 MHz ^1^H Larmor frequency with 256* and 1536* complex pairs in the indirect ^15^N and direct ^1^H dimensions with corresponding acquisition times of 33 ms and 57 ms and sweep widths of 45 and 16 ppm centered at 116 and 4.7, respectively. ^15^N and ^1^H chemical shifts were measured from the cross peaks on the HSQCs for the given mixture of components. Chemical shift deviations for ^1^H and ^15^N were quantified by subtracting the chemical shift of the reference spectrum (1:0 sample for titrations) from the respective value of each other sample (for the individual RRMs compared to 1–189, 1–189 was the reference spectrum). The average chemical shift deviations were calculated by the equation: $\Delta \delta ({}_{}^{15}{\rm N},\ \ {}_{}^1{\rm H}) = \ \sqrt {\frac{1}{2}(\Delta \delta {{\rm H}^2} + ( {0.14\Delta \delta {\rm N}{)^2}} )}$.

#### Relaxation

Motions of the backbone of hnRNPA2 1–189 were probed using ^15^N *R*_1_, temperature-compensated ^15^N *R*_2_, and heteronuclear NOE experiments using standard pulse sequences (hsqct1etf3gpsitc3d, hsqct2etf3gpsitc3d, hsqcnoef3gpsi, respectively, from Topspin 3.5, Bruker). Interleaved experiments comprised 256 × 1536 total points in the indirect ^15^N and direct ^1^H dimensions, respectively, with corresponding acquisition times of 33 and 57 ms, sweep width of 45 and 16 ppm, centered at 116 and 4.7 ppm, respectively. ^15^N *R*_2_ experiments had an interscan delay of 2.5 s, a Carr-Purcell-Meiboom-Gill (CPMG) field of 556 Hz, and total *R*_2_ relaxation CMPG loop-lengths of 16.5, 148.5, 115.5, 33, 66, 82.5 and 49.5 ms at 850 MHz ^1^H frequency and 16.5, 247.5, 115.5, 33, 148.5, 66 and 198 ms at 500 MHz ^1^H frequency. ^15^N *R*_1_ experiments had an interscan delay of 1.2 s at 850 MHz ^1^H frequency and 1.5 s at 500 MHz ^1^H frequency, and total *R*_1_ relaxation loop-lengths of 100, 1500, 200, 800, 1200, 600 and 400 ms at 850 MHz ^1^H frequency and 100, 1200, 200, 800, 300, 600 and 400 ms at 500 MHz ^1^H frequency. Heteronuclear NOE experiments were conducted with an interscan delay of 7 s at 850 MHz ^1^H frequency and 5 s at 500 MHz ^1^H frequency. τ_c_ was calculated by averaging the *T*_1_ and *T*_2_ from each residue for the apo and bound form. These values were then used in the equation ${\tau _c} = \ \frac{1}{{4\pi {\nu _{\rm{N}}}}}\sqrt {6\frac{{{T_1}}}{{{T_2}}} - 7}$ where *ν*_N_ is the ^15^N resonance frequency in Hz. Predicted *τ*_c_ was calculated using http://nickanthis.com/tools/tau (empirical formula).

#### Residual dipolar coupling

Backbone amide (^1^*D*_NH_) RDCs for well resolved ^1^H–^15^N cross-peaks were measured on samples of weakly aligned hnRNPA2 1–189 in a dilute liquid crystalline medium of PEG-hexanol at 850 ^1^H MHz frequency at 298 K using a standard pulse sequence (hsqcf3gpiaphwg.2 from TopSpin 3.5, Bruker). Samples consisted of 200 μM hnRNPA2 1–189 in 20 mM NaPi pH 6.8 150 mM NaCl with 10% D_2_O in PEG-hexanol (4% PEG by weight) with an r value of 0.96 (mol PEG/mol hexanol). Interleaved experiments comprised of 384 × 2048 total points in the indirect ^15^N and direct ^1^H dimensions, respectively, with corresponding acquisition times of 131 ms and 86 ms, sweep width of 34 and 14 ppm, centered at 116 and 4.7 ppm, respectively. To avoid structural noise from flexible regions, only the backbone amides from secondary structure elements were included in the analysis. Singular value decomposition of the experimental ^1^*D*_NH_ RDCs were fit to the coordinates of the isolated RRM1 and RRM2 of the 5HO4 crystal structure of hnRNPA2 RRMs as well as the full length tandem RRMs (15).

## RESULTS

### Apo hnRNPA2 1–189 is composed of two flexible RRM domains

**Figure 1.**
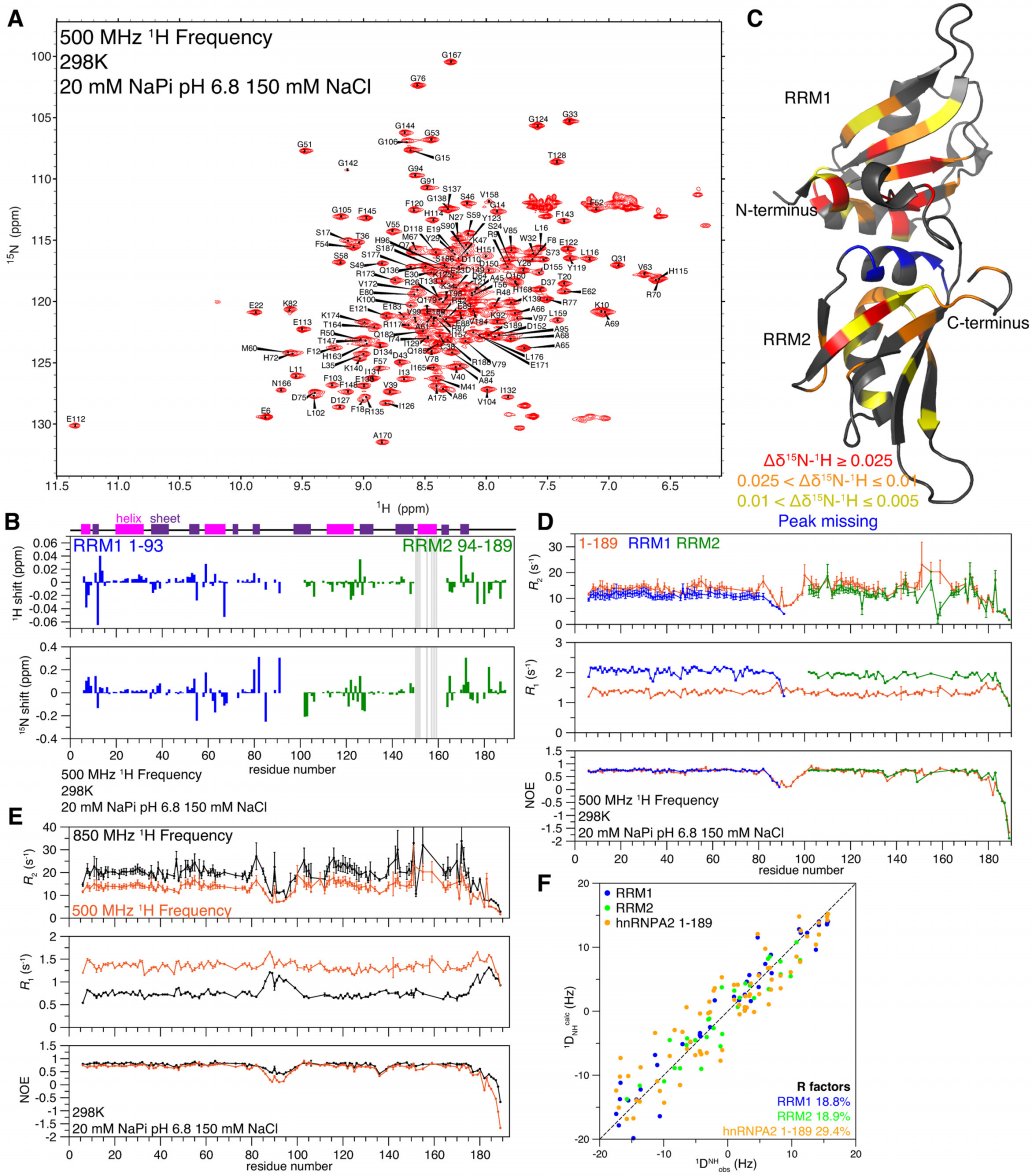
hnRNPA2 RRMs remain folded and interact in the absence of RNA but do not form a rigid structure. See also Supplementary Figure S1. (**A**) ^1^H–^15^N HSQC of hnRNPA2 1–189 is consistent with a folded protein. (**B**) Chemical shift deviations for the individual RRMs compared to 1–189 are small and primarily at the N- and C-termini of the constructs (e.g. due only to primary structure differences), except in RRM2 where shifts were too large to determine peak identity by overlay, as indicated by grey bars. Secondary structure elements as determined by NMR (see Supplementary Figure S1A) shown at top, α-helices in magenta, β-sheets in purple. (**C**) Average chemical shift deviations (plotted in Supplementary Figure S1C) of difference between individual RRM spectra and tandem RRM spectra mapped on the structure of hnRNPA2 RRMs (PDB: 5HO4) to show where RRM1 and RRM2 interact with each other in the absence of RNA. (**D**) NMR spin relaxation parameters ^15^N *R*_2_, ^15^N *R*_1_, and hetNOE values for 1–189 (orange), RRM1 (1–93 blue), and RRM2 (94–189 green) at 500 MHz ^1^H frequency indicate the RRM move similarly but have slowed tumbling when the RRMs are linked. (**E**) NMR spin relaxation parameters ^15^N *R*_2_, ^15^N *R*_1_ and hetNOE values for 1–189 at 850 MHz ^1^H frequency (black) and 500 MHz ^1^H frequency (orange) indicate the presence of a flexible linker between the individual RRMs. (**F**) Agreement between the experimental (^1^*D*_NH_^obs^) RDCs and the back calculated (^1^*D*_NH_^calc^) RDCs from PDB 5HO4 (15) for RRM1 (blue) and RRM2 (green) alone show lower R factors (better agreement) than for the tandem RRMs together (orange). See Table 2.

We first used NMR to examine the unbound (apo) form of the RRMs of hnRNPA2 (residues 1–189). The two-dimensional ^1^H–^15^N heteronuclear single-quantum coherence (HSQC) spectrum of hnRNPA2 1–189 has a wide chemical shift dispersion, consistent with a folded protein (Figure 1A). Secondary structure metrics derived from the observed chemical shifts are consistent with a protein containing both α-helices and β-sheets (Supplementary Figure S1A) and the location of these elements are consistent with the published structure (15). HSQC spectra of the isolated RRMs (RRM1 1–93, RRM2 94–189) show substantial, but incomplete, overlap with the HSQC of the tandem RRMs (hnRNPA2 1–189) (Supplementary Figure S1B). Quantification of the chemical shift deviations between the individual RRMs and 1–189 show some regions of increased chemical shift difference, including the N- and C-termini of both constructs (Figure 1B). There are also some positions far from the termini, including a contiguous segment in RRM2 (residues 150–163) where shifts were so large that we were unable to transfer assignments by overlay. These chemical shift deviations suggest there may be some significant interactions between the individual RRMs in 1–189 as was found for hnRNPA1 (16). We mapped the average chemical shift deviation (Figure 1C, Supplementary Figure S1C) onto the published crystal structure of the hnRNPA2 RRMs (15) and found that the largest shifts are distributed throughout the structure, but many localize to the folded regions of the protein, particularly the helix at the back of RRM2, suggesting that this region may mediate weak intramolecular interactions (Figure 1C). To test this model, we performed titrations mixing the individual RRMs and found larger chemical shifts mixing RRM1 into ^15^N RRM2 than mixing RRM2 into ^15^N RRM1 (Supplementary Figure S1D), suggesting that there may be some weak interactions between the individual RRMs.

We performed NMR spin relaxation experiments *R*_2_, *R*_1_ and hetNOE to examine the local reorientational motions at each backbone position (Figure 1D). Differences observed in *R*_2_ and *R*_1_ are due to the decreased size of the individual RRMs compared to the tandem RRM construct, but all are consistent with each RRM being a single, folded domain (Figure 1D). NMR spin relaxation experiments for 1–189 at a second ^1^H Larmor frequency are largely similar (Figure 1E). The apparent τ_c_ values for apo hnRNPA2 1–189 at two temperatures are smaller than what is predicted for a protein of that molecular weight (Table 1), suggesting that the RRM1 and RRM2 can move independently and are not rigidly confined in the apo state, unlike the bound state (see below). In contrast, the apparent τ_c_ values for the individual RRMs are in fact larger than what is predicted for proteins of those molecular weights, consistent with folded domains with disordered tails that slow tumbling. These findings are in contrast to what was found for hnRNPA1 individual RRMs and tandem RRMs, which have much higher τ_c_ values (16).

**Table 1.** Predicted and calculated τ_c_ values indicate apo hnRNPA2 1–189 is not rigid

	Temperature (°C)	Molecular weight (Da)	τ_c_ predicted (ns)	τ_c_ calculated (ns)
Apo hnRNPA2 1–189	37	21 854	9.91	7.95
hnRNPA2 1–189 + rA2RE11	37	25 381	11.49	11.85
Apo hnRNPA2 1–189	25	21 854	13.28	10.57
Apo hnRNPA2 1–93	25	10 785	6.64	7.8
Apo hnRNPA2 94–189	25	11 411	7.01	7.4

To further probe if RRM1 and RRM2 adopt a rigid arrangement in solution, we performed residual dipolar coupling NMR experiments sensitive to global orientation on apo hnRNPA2 1–189. Singular value decomposition fitting of the experimental ^1^*D*_NH_ RDCs to the coordinates of the isolated RRM1 and RRM2 of the 5HO4 crystal structure of hnRNPA2 bound to RNA (resolution 1.85 Å) (15) yields RDCs R-factors (24) of 18.8% and 18.9%, respectively (Figure 1F and Table 2), confirming that the tertiary fold of the secondary structure elements within RRM1 and RRM2 is unchanged relative to the crystal structure. When the RDC data are fit to coordinates of hnRNPA2 1–189 (i.e. RRM1 and RRM2 together), the RDC *R*-factor increases by more than 50% (*R*-factor ∼29.4%, Table 2). These data demonstrate that, in solution, the relative orientation between the RRM1 and RRM2 differs from the ones observed in the crystal structure of hnRNPA2 in complex with nucleic acids (25), which is consistent with NMR relaxation data suggesting fast and at least partially independent rotational diffusion for the structural domains of apo hnRNPA2.

**Table 2.** RDC analysis

		Euler angles (°)^a^					
	Number of RDCs	*ϕ*	*θ*	*ψ*	*D_a_* (Hz)^a^	*η* ^a^	*R*-factor (%)^b^
RRM1	40	93	78	95	10.6	0.23	18.8
RRM2	31	189	68	94	9.2	0.55	18.9
hnRNPA2 1–189	71	97	82	108	9.1	0.47	29.4

^a^The alignment tensor is described by five parameters: three Euler angles (*ϕ*, *θ* and *ψ*), the magnitude of the alignment tensor *D_a_*, and the rhombicity *η*. The fact that the values of the alignment tensors of the N- and C-terminal domains are substantially different indicates that their relative orientation is solution differs from the one observed in the crystal structure of apo hnRNPA2.

^b^The RDC *R*-factor is given by 100 × [〈(*D*_obs_ – *D*_calc_)^2^〉/(2〈*D*_obs_〉^2^]^1?2^, where *D*_obs_ and *D*_calc_ are the observed and calculated RDCs, respectively (24).

### hnRNPA2 RRMs bind the A2RE weakly

**Figure 2.**
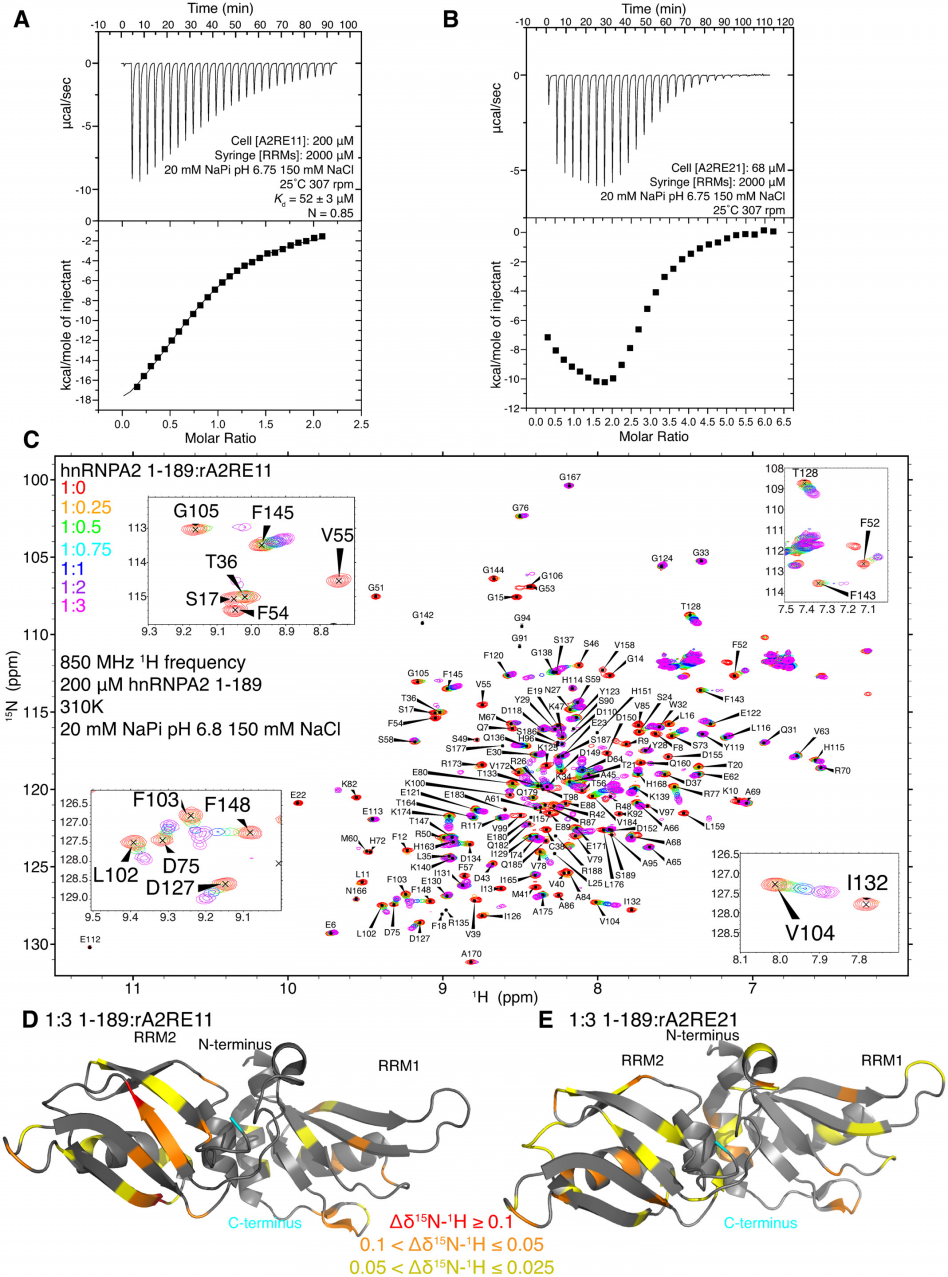
hnRNPA2 1–189 weakly binds the A2RE RNA. See also Supplementary Figure S2. (**A**) ITC of the hnRNPA2 1–189 binding to rA2RE11. Dissociation constant is about 52 μM and the interaction stoichiometry is closest to 1 to 1. (**B**) ITC of hnRNPA2 1–189 binding to rA2RE21. The isotherm has a distinctive biphasic curve and inflection point at or above 2.0, consistent with multiple binding site scheme (i.e. at least, and most likely, two hnRNPA2 1–189 can simultaneously bind rA2RE21). (**C**) ^1^H–^15^N HSQC of hnRNPA2 1–189 titrated with increasing concentrations of rA2RE11. Data is consistent with weak binding with most peaks being in intermediate-fast exchange. Insets show a number of peaks that shift (e.g. T128, F145, V104), decrease signal intensity (e.g. I132, F54), or shift and decrease then increase signal intensity (e.g. G105, F143). (**D**) Average chemical shift deviations for the titration of rA2RE11 (from S3A-B) plotted on PDB structure of hnRNPA2 RRMs 5HO4. (**E**) Average chemical shift deviations for the titration of rA2RE21 (from S3D–E) plotted on 5HO4.

As hnRNPA2 is reported to have a high affinity interaction with the A2RE (5), we performed isothermal titration calorimetry (ITC) with the A2RE. We first performed ITC on the 11mer RNA (rA2RE11), which is the minimal sequence that binds hnRNPA2 (6). With rA2RE11 and hnRNPA2 1–189, we found a *K*_d_ of about 52 μM (Figure 2A), much weaker than the reported value of about 50 nM (5). We repeated this experiment at other concentrations of hnRNPA2 and A2RE11 and found similarly weak *K*_d_ values (Table 3). Thinking that this low value may be unique to the buffer conditions used (20 mM NaPi pH 6.75 150 mM NaCl), we re-did the experiment in a potassium phosphate buffer at the same pH with no salt and found a similar value of 35 μM (Supplementary Figure S2A). Of note, the calculated ratio of 1–189 to rA2RE11 for these experiments is about one to one (Figure 2A, Supplementary Figure S2A, Table 3), suggesting that one rA2RE11 is capable of binding the tandem RRMs. To be sure heat of dilution of hnRNPA2 1–189 was not altering our results, we performed ITC diluting hnRNPA2 1–189 into buffer (i.e. no RNA) and observe nearly no heat signal (Supplementary Figure S2B). To confirm that we were able to detect tight binding of hnRNPA2 1–189 to any RNA sequence, we performed ITC on the 10mer sequence used by Wu *et al.* (15). The interaction between hnRNPA2 1–189 and the Wu_10mer RNA showed a binding affinity of 228 ± 2 nM (Supplementary Figure S2C), similar to the reported value and about two orders of magnitude tighter than any binding affinity we observed with A2RE, further suggesting that our data showing that the rA2RE11 interacts weakly with hnRNPA2 1–189 are reliable.

**Table 3.** Additional 1–189 – rA2RE11 ITC experiments

Syringe: [hnRNPA2 1–189] (μM)	Cell: [rA2RE11] (μM)	N	*K*_d_ (μM)
7000	800	0.921	139 ± 10
7000	800	0.798	143 ± 10
4600	115	0.785	19.8 ± 0.4

Next, we performed ITC with rA2RE21, the full A2RE present in myelin basic protein mRNA, to determine if it can bind two copies of hnRNPA2 1–189. At conditions where hnRNPA2 1–189 shows simple 1:1 binding with rA2RE11, we obtained a biphasic curve when hnRNPA2 1–189 binds rA2RE21 (Figure 2B). This curve cannot be explained by a single binding event and transition near 2.0 molar ratio indicates that at least two molecules of hnRNPA2 1–189 are involved. Although the data do not uniquely specify parameters for a more complicated binding model, this biphasic curve is consistent with multiple binding events, indicating that two hnRNPA2 1–189 molecules are likely able to bind the rA2RE21. To confirm this result, we repeated the experiment at a different concentration of rA2RE21 and obtained a similar curve (Supplementary Figure S2D). We further measured the binding of a scrambled rA2RE21, rA2RE21^scr^, which has the same nucleotide composition but scrambled sequence, to test the hypothesis that the rA2RE11 has sequence specificity. We found signatures of significantly reduced binding to rA2RE21^scr^ (Supplementary Figure S2E), demonstrating that although the hnRNPA2 binding affinities to A2RE RNA are weak, they are sequence specific.

To understand how binding the shorter A2RE11 affects hnRNPA2 1–189, we performed an NMR HSQC titration of the RRMs with increasing amounts of rA2RE11 (Figure 2C). Some resonances shift and broaden beyond detection at high RNA concentration, some resonances shift and broaden at intermediate concentrations but partially re-sharpen at the highest concentrations, and some resonances appear to shift without significant broadening (Figure 2C). These observations are suggestive of chemical exchange on the fast/intermediate chemical shift timescale and are consistent with the weak binding we found using ITC. A large proportion of the broadening appears to arise from chemical exchange on the intermediate timescale, as peaks that look to primarily shift also decrease in intensity with addition of rA2RE11. Chemical shift perturbations (CSPs) tend to increase with increasing rA2RE11 concentration (Supplementary Figure S3A). Importantly, the chemical shifts appear to approach saturation at the highest concentration of rA2RE11 (1:3 protein:RNA where the protein is 200 μM and the RNA is 600 μM), consistent with the ∼50 μM dissociation constant and 1:1 binding stoichiometry measured by ITC. We plotted the ^1^H–^15^N weighted average CSPs (Supplementary Figure S3B) for the highest concertation of rA2RE11 on the structure of the RRMs and found that most of the large CSPs are on the second RRM and the majority of the large (>0.025 ppm) average shifts are on the β-sheet faces of the RRMs (Figure 2D), which is consistent with how hnRNPA2 binds other RNAs (15). Interestingly, the peak intensities broaden and almost none recover to their initial intensity, consistent with a slowing in the tumbling rate of the molecule upon binding (26).

We also performed a similar titration with rA2RE21 and found similar increasing CSPs with rA2RE21 concentration (Supplementary Figure S3C). The peak intensities of 1–189 also tend to broaden then partially recover with increasing rA2RE21 concentration, although they do not recover to the same extent as they do with the rA2RE11, likely due to the larger size of rA2RE21, which further slows tumbling (Supplementary Figure S3D). The average CSP for the titration with rA2RE21 was similar to the ones found with rA2RE11 (Figures 2E, Supplementary Figure S3E). No direct evidence of simultaneous binding of two copies of hnRNPA2 1–189 with the longer rA2RE21 is observed here, likely because of the weak binding affinity and, at high RNA concentrations, excess RNA such that each protein molecule binds a different RNA. As hnRNPA2 binds DNA as well as RNA, we also performed a titration with a single stranded DNA oligomer of the A2RE11 (dA2RE11) and found that most peaks broaden and did not re-sharpen at excess dA2RE11 concentrations (Supplementary Figure S3F), unlike rA2RE11, consistent with even weaker binding of hnRNPA2 1–189 to DNA than to RNA.

Finally, we performed experiments to determine the NMR spin relaxation parameters on apo and rA2RE11-bound hnRNPA2 1–189. The increased ^15^N *R*_2_ and decreased ^15^N *R*_1_ observed in the 1:3 hnRNPA2 1–189:rA2RE11 sample compared to the apo sample are indicative of the formation of a higher molecular weight complex, as is expected by binding of the RNA oligomer to 1–189 (Supplementary Figure S3G). The hetNOE is almost identical for the bound and apo forms (Supplementary Figure S3G), suggesting that the local flexibility is not significantly changed by RNA-binding. Of note, the loop connecting RRM1 and RRM2 remains disordered in the RNA-bound form. However, the global tumbling is different between the apo and bound forms of hnRNPA2 1–189. In contrast to the apo RRMs, the hnRNPA2 1–189 bound to rA2RE11 has an apparent τ_c_ consistent with its molecular weight (Table 1). This observation combined with the observation that the apo RRMs (hnRNPA2 1–189) have a smaller τ_c_ than predicted suggest that RRM1 and RRM2 are not rigidly confined in the apo state, but they are in the bound state. Taken together, binding the minimal rA2RE11 locks the RRMs of hnRNPA2 into position and the complete rA2RE21 creates a site for binding two copies of hnRNPA2.

### hnRNPA2 RRM2 binds calcium

**Figure 3.**
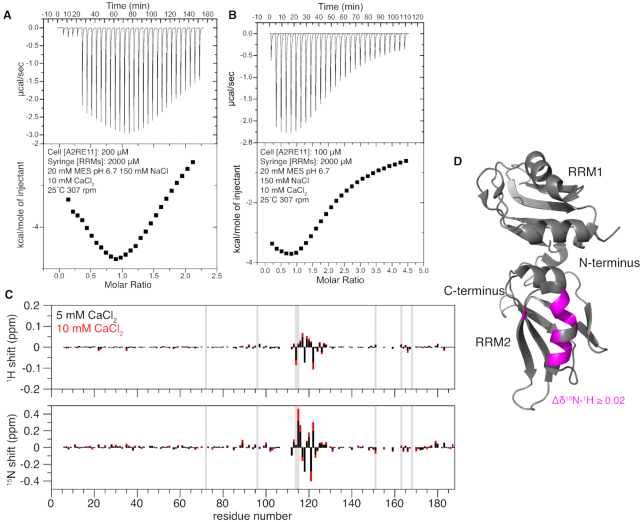
hnRNPA2 RRM2 binds calcium ions. (**A**) ITC of the RRMs binding to rA2RE11 in the presence of calcium. The isotherm has a biphasic curve consistent with multiple binding, indicating that the presence of calcium is altering the binding of the RRMs to the RNA, although the apparent binding affinity does not increase. (Note that the small peaks in the beginning of the thermogram were caused by a series of 5 small (2 μl) injections to ensure reliable data for the first large injection used for isotherm quantification). (**B**) ITC of the RRMs binding to rA2RE11 in the presence of calcium at half the concentration of RNA. The isotherm still has a biphasic curve consistent with multiple binding events, indicating that the presence of calcium is altering the binding of the RRMs to the RNA. (**C**) Chemical shift deviation of hnRNPA2 RRMs with 5 or 10 mM CaCl_2_ added. Chemical shift deviations indicate the presence of a binding pocket for calcium in the second RRM around residues 110–125. Vertical grey bars indicate histidine residues, showing that the shifts are not localized to histidine positions and therefore unlikely to be due to possible slight deviations in pH. (**D**) Average CSPs for the 10 mM CaCl_2_ condition plotted onto 5HO4 suggest that Ca^2+^ primarily binds a single helix at the back of RRM2.

As hnRNPA2 has been reported to bind RNA in a calcium dependent manner (27), we performed ITC with rA2RE11 in a buffer containing 10 mM calcium. The presence of calcium did not increase the affinity of 1–189 for rA2RE11, however we observed a biphasic curve for two concentrations of rA2RE11, consistent with multiple binding events (Figure 3A-B). Finding this result curious, we performed an NMR titration of hnRNPA2 1–189 and CaCl_2_, to determine if hnRNPA2 1–189 is able to bind calcium. Indeed, we found that approximately residues 110–125 specifically bind Ca^2+^ (Figure 3C, D). This suggests a mechanism by which calcium may alter RNA binding, as part of RRM2 is occupied binding calcium, which may disrupt RNA binding.

### Binding of A2RE prevents *in vitro* phase separation and aggregation of hnRNPA2

**Figure 4.**
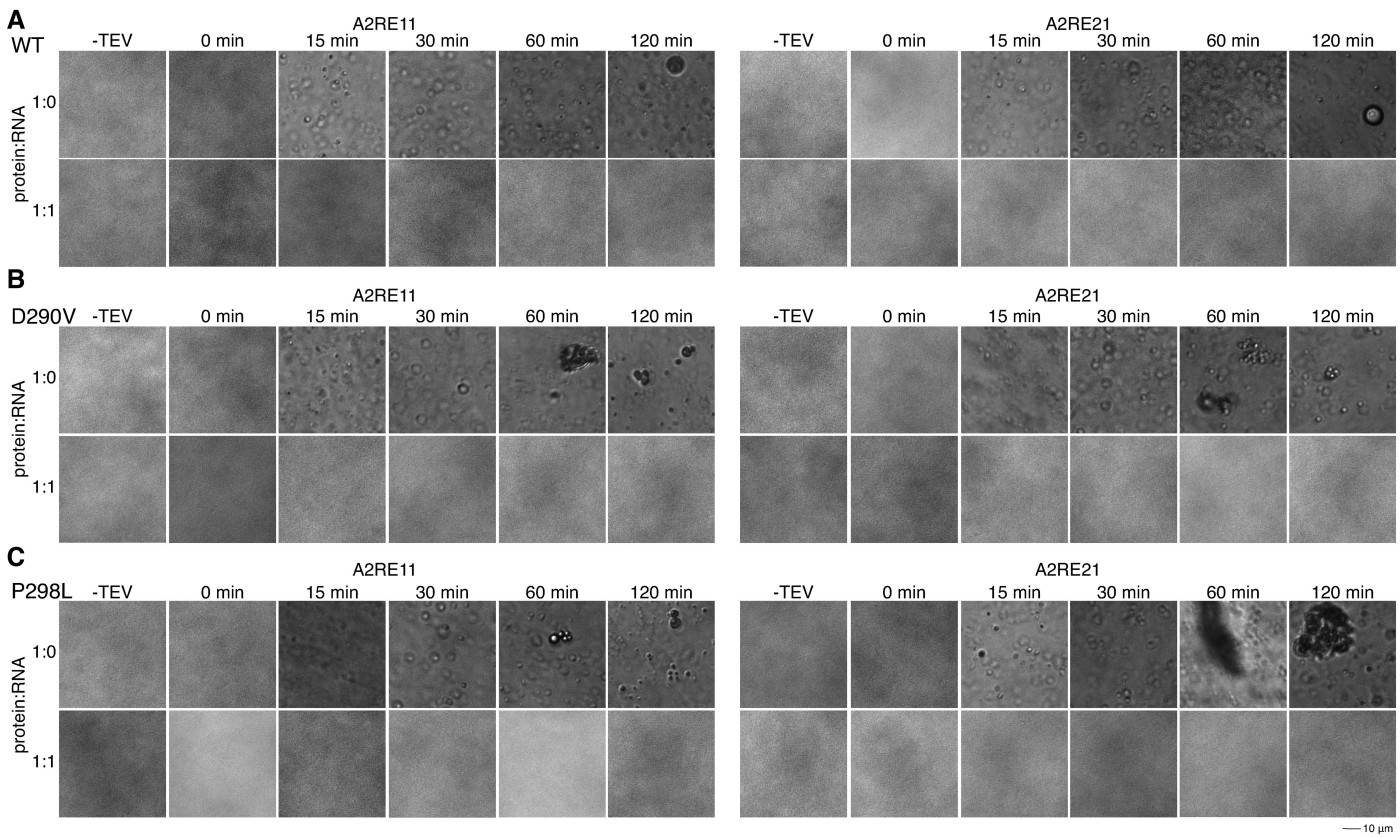
rA2RE11 and rA2RE21 prevent phase separation and aggregation of full-length hnRNPA2 WT and mutants. See also Supplementary Figure S4. (**A**) After cleavage of a C-terminal maltose binding protein solubility tag by TEV protease, hnRNPA2 FL WT undergoes LLPS. In the presence of either rA2RE11 or rA2RE21, hnRNPA2 FL WT does not undergo LLPS. (**B, C**) After cleavage of a C-terminal maltose binding protein solubility tag by TEV protease, hnRNPA2 FL D290V (B) and P298L (C) undergo LLPS followed by aggregation. In the presence of either rA2RE11 or rA2RE21, neither phase separation nor aggregation occur.

As hnRNPA2 can undergo LLPS (10) and RNA has been shown to alter LLPS of other proteins (28–32), we tested whether A2RE RNA alters LLPS of hnRNPA2. As there are two binding sites for hnRNPA2, we hypothesized that rA2RE21 would increase phase separation of hnRNPA2 by bringing two hnRNPA2 molecules in close proximity (forming a 2:1 protein:RNA complex), allowing their LCs to interact and phase separate. In contrast, we expected that rA2RE11 would not increase LLPS as it only contains one hnRNPA2 binding site (forming a 1:1 protein:RNA binding site). However, at all conditions tested (two salt concentrations, four ratios of protein to RNA, both length RNA oligomers), addition of either rA2RE11 or rA2RE21 prevented LLPS of full-length hnRNPA2 WT (Supplementary Figure S4A-C). We saw no evidence that addition of RNA simply delayed formation of visible droplets, as no hnRNPA2 droplets were observed in the presence of RNA at any time tested, up to two hours, though droplets were observed at all time points tested in the absence of RNA (Figure 4A). Although weak multivalent interactions are known to contribute to LLPS (21), it is important to note that, given the relatively weak affinities for the A2RE with hnRNPA2 1–189 (i.e. the LLPS assay is performed at concentrations below the dissociation constant), a majority of the RNA is not predicted to bind two copies of the protein simultaneously, perhaps contributing to the lack of stimulation of LLPS by addition of rA2RE21. Here, we performed all LLPS assays at 15 μM hnRNPA2 FL as hnRNPA2 FL undergoes robust LLPS at that concentration (10,33). Despite the fact that at these concentrations (given the binding affinities we found for rA2RE11 and rA2RE21) only a fraction of hnRNPA2 RRMs would be expected to bind RNA, we do see a profound disruption of LLPS by addition of RNA on LLPS. Of note, it is unclear what the ratio of RNA to protein molecules is in granules in cells, so it is possible that there is an even greater ratio of protein:RNA in those granules, which may explain how granules maintain their integrity in cells.

To further understand this surprising finding, we performed similar experiments on additional RNA sequences. First, we asked if the LC of hnRNPA2 can directly interact with RNA and hence contribute to the inhibition of LLPS. To this end, we obtained a Cy-3 labeled rA2RE11 and mixed it at varying ratios with hnRNPA2 LC at conditions where hnRNPA2 LC undergoes LLPS (10). Indeed, we found that this Cy-3 labeled rA2RE11 avidly partitions into hnRNPA2 LC droplets (Supplementary Figure S4D). As such, we hypothesized that a longer RNA may stimulate hnRNPA2 full-length LLPS in our original assay as both the RRMs and LC could simultaneously interact with RNA. We obtained two 60-mer RNAs, one containing the A2RE21 sequence flanked by myelin basic protein mRNA sequence (long_rA2RE21) and one with the same flanking sequence but the scrambled A2RE (long_rA2RE21^scr^) that does not specifically bind the RRMs (see Supplementary Figure S2E), and performed LLPS assays with hnRNPA2 FL. In contrast to our hypothesis, we once again observed no LLPS in the presence of any RNA concentration for both 60mers (Supplementary Figure S4E). Further, the rA2RE21^scr^ alone also eliminates LLPS of hnRNPA2 (Supplementary Figure S4F), suggesting that the effect observed is not due to binding the RRMs with sequence specificity. Finally, we tested whether the tight-binding Wu_10mer RNA altered LLPS of hnRNPA2 FL. In contrast to all other RNAs tested, the Wu_10mer RNA did not eliminate LLPS of hnRNPA2 FL at low concentrations (1:0.25 and 1:0.5 protein:RNA ratios) but does suppress LLPS at 1:1 ratio and above (Supplementary Figure S4G). As such, it seems that at these conditions the LC domain of hnRNPA2 mediates contacts leading to LLPS in the absence of RNA but competes with the RRMs for RNA binding, suggesting that RNA interaction with hnRNPA2 LC is important for altering *in vitro* LLPS.

After observing the complete elimination of LLPS of hnRNPA2 at all rA2RE11 or 21 concentrations tested, we hypothesized that addition of RNA would also prevent aggregation of the hnRNPA2 disease-associated mutants, D290V and P298L. Indeed, while both D290V and P298L formed aggregates over time, the addition of RNA prevented formation of both liquid droplets and solid aggregates at these conditions (Figure 4B, C). This prevention of aggregation was even greater than that exerted by partial tyrosine phosphorylation (33), as here neither D290V nor P298L showed any droplets in the presence of RNA.

## DISCUSSION

**Figure 5.**
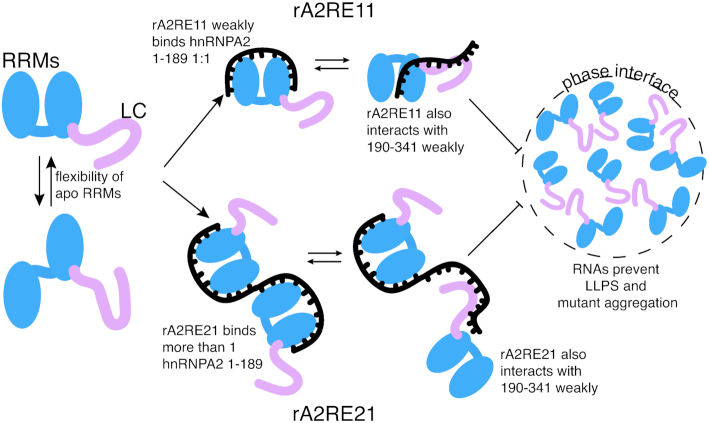
Model. Apo hnRNPA2 1–189 has some conformational flexibility in the orientation of the two RRM domains relative to one another. hnRNPA2 1–189 can bind one rA2RE11 molecule, which locks the relative orientation of the RRMs, while more than one molecule of hnRNPA2 1–189 can bind one rA2RE21. The LC of hnRNPA2 competes with the RRMs for interaction with RNA, preventing LC–LC interactions critical for LLPS. Addition of RNA prevent LLPS of full length hnRNPA2 and aggregation of disease mutant hnRNPA2 by binding to the LC domain.

Here, we examine the structural basis for the interaction between the hnRNPA2 RRMs (1–189) and the A2RE of myelin basic protein mRNA. In contrast to previous reports (5), we find that both the 11mer and 21mer RNA oligomers bind to hnRNPA2 1–189 weakly (Figure 5), with a binding affinity on the order of tens of micromolar. Given that we have recently shown hnRNPA2 is highly prone to phase separate and aggregate and requires careful use of solubility tags and RNA removal to prevent pre-aggregation (10,22,30,33), it is possible that self-interactions between full-length hnRNPA2 molecules (e.g. pre-formed aggregates) and protein-surface interactions complicated previous bio-sensor measurements performed with full-length recombinant protein. We did not observe a significant difference in binding affinity in the presence of calcium or using a potassium phosphate buffer without sodium chloride, suggesting that for this construct and the A2RE RNA, the interaction is not primarily electrostatic. Indeed, recognition of the bases, not the phosphodiester backbone, is the common mechanism for RRM-RNA interaction (34). Importantly, we can recapitulate tighter binding of our hnRNPA2 construct with a different RNA previously shown to interact with hnRNPA2 (15). We also observed a weaker though comparable interaction with DNA oligos, suggesting that hnRNPA2 may interact with both RNA and DNA in cells; hnRNPA2 is known to be involved in the maintenance of telomeres (35). The regions of hnRNPA2 observed to interact with RNA in the crystal structure are consistent with the chemical shifts observed with RNA in our NMR studies. In contrast to the published crystal structure of hnRNPA2 bound to other RNA sequences (15), the interaction between 1 and 189 and rA2RE11 requires only one molecule of hnRNPA2 and one molecule of rA2RE11, suggesting the observation that the individual RRMs of a single hnRNPA2 molecule bind to separate RNA molecules is not the case for the A2RE11 and may be a crystal artifact (14). Furthermore, we saw no evidence for a significant change in the apparent dissociation constant at different concentrations of RNA (the fixed concentration in the ITC cell) which would be expected for a two RNA to two protein binding mode. Hence, these findings highlight the importance of solution-state experiments for fully understanding binding modes.

In contrast to our hypothesis, we observed that addition of either rA2RE11 or rA2RE21 completely abolished the ability of full length hnRNPA2 to undergo LLPS, while we expected the 21mer to stimulate LLPS as the RNA is multivalent (Figure 5). This elimination of LLPS may be for a few reasons. First, full length hnRNPA2 is more soluble in higher salt conditions (as evidenced by Supplementary Figure S4C and our purification conditions of 1 M NaCl). RNA is a highly charged molecule and adding a short RNA may have a similar effect as increasing the salt concentration of the sample. Second, the low complexity (LC) domain of hnRNPA2 has multiple RGG motifs, which have been shown to interact with RNA in other proteins (36–38) and the LC interacts with A2RE11 RNA in a liquid-like phase. It seems that any LC–RNA interactions in the context of the full-length protein contribute to elimination of LLPS, suggesting that LC-LC interactions are crucial for maintaining the integrity of the droplets. Interestingly, other groups have found that short RNAs do not stimulate phase separation of G3BP1 while longer RNAs do (12,13), suggesting that RNA length plays a larger role in providing multivalency than we expected. However, with 60-mer RNA oligomers, we still observed no LLPS, suggesting the protein-protein interactions are more important for LLPS than RNA-protein interactions for hnRNPA2 at these conditions. The impact of physiological arginine methylation, which alters hnRNPA2 LC self-interaction and LLPS (10), on hnRNPA2 LC interactions with RNA will be important to study in the future. Though our focus here is on the RNA-binding properties of the hnRNPA2 RRMs to the A2RE, it is possible that the *in vitro* LLPS experiments may not represent the effect of RNA on hnRNPA2 phase separation in cells where other protein and RNA interactions may cooperate with the A2RE interaction with hnRNPA2 RRMs to stabilize a granule (33).

We also observed that aggregation of mutant full-length hnRNPA2 was abolished upon addition of RNA. Interestingly, this finding parallels work showing that RNA is excluded from TDP-43 inclusions in cells and that total HEK298 cell RNA reduced TDP-43 phase separation and aggregation *in vitro* (39). Further, reduction of TDP-43 assembly in neurons by short oligonucleotides (34 nucleotides long) prevented neurotoxicity (39), suggesting that the same may be true for hnRNPA2. hnRNPA2 is also a component of transport granules, membraneless organelles that move mRNAs from the perinucleus to sites of local translation (2). While these granules are hypothesized to be phase separated (21,33), that has not yet been tested in cells. However, transport granules contain multiple components besides RNA and hnRNPA2 that may contribute to the multivalent interactions required for LLPS.

## DATA AVAILABILITY

NMR chemical shift assignments for hnRNPA2 1–189 at 298 K (BMRB: 50254), 310 K (BMRB: 50255) and bound hnRNPA2 1–189 at 310 K (BMRB: 50257) can be obtained online from the Biological Magnetic Resonance Database (BMRB, http://www.bmrb.wisc.edu/). Plasmids generated herein can be found at Addgene.org.

## Supplementary Material

gkaa710_Supplemental_File
