# Supplementary material for: Weak binding to the A2RE RNA rigidifies hnRNPA2 RRMs and reduces liquid–liquid phase separation and aggregation
Source: Nucleic Acids Res. 2020 Sep 1;48(18):10542–54. doi: 10.1093/nar/gkaa710 (PMC7544213; doi:10.1093/nar/gkaa710)
Supplement: gkaa710_Supplemental_File [file gkaa710_supplemental_file.pdf]

Supplemental Figures

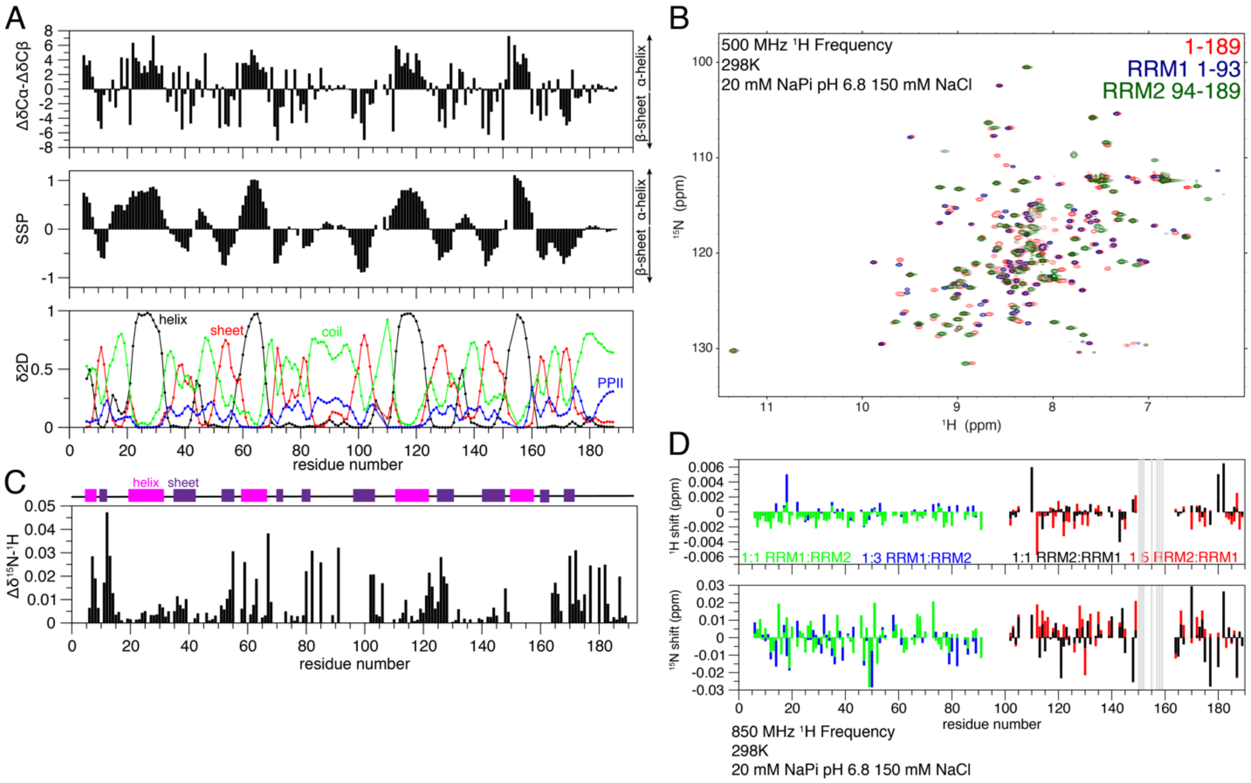

<u>Figure S1: Structure and interactions between RRM1 and RRM2 of hnRNPA2 1-189. Related to Figure 1.</u>

A) The secondary chemical shifts (ΔδCα- ΔδCβ), secondary structure prediction (SSP), and δ2D predicted secondary structure propensity of hnRNPA2 1-189 are all consistent with a folded protein consisting of both α-helices and β-sheets.

B) Overlay of ^{1}H-^{15}N HSQCs of 1-189 (RRM1+RRM2), 1-93 (RRM1), and 94-189 (RRM2) indicate that there are no global differences in structure between the individual RRMs and the full construct. Secondary structure elements (reproduced from Figure 1B) are indicated at top, magenta = α-helices, purple = β-sheets.

C) Average chemical shift deviations from S1C plotted on PDB structure 5HO4 of hnRNPA2 RRMs. There may be a transient interaction between the RRMs in the apo form, particularly the helix in RRM2 corresponding to the residues that move too far to be identified by HSQC overlay (blue).

D) "*In trans*" titrations of the individual RRMs show minor chemical shift perturbations in similar regions to (E), suggesting that the RRMs interact with each other, although more strongly when they are connected ("*in cis*"). Peaks that shifted too much in the individual RRM spectra to be identified are indicated with grey bars.

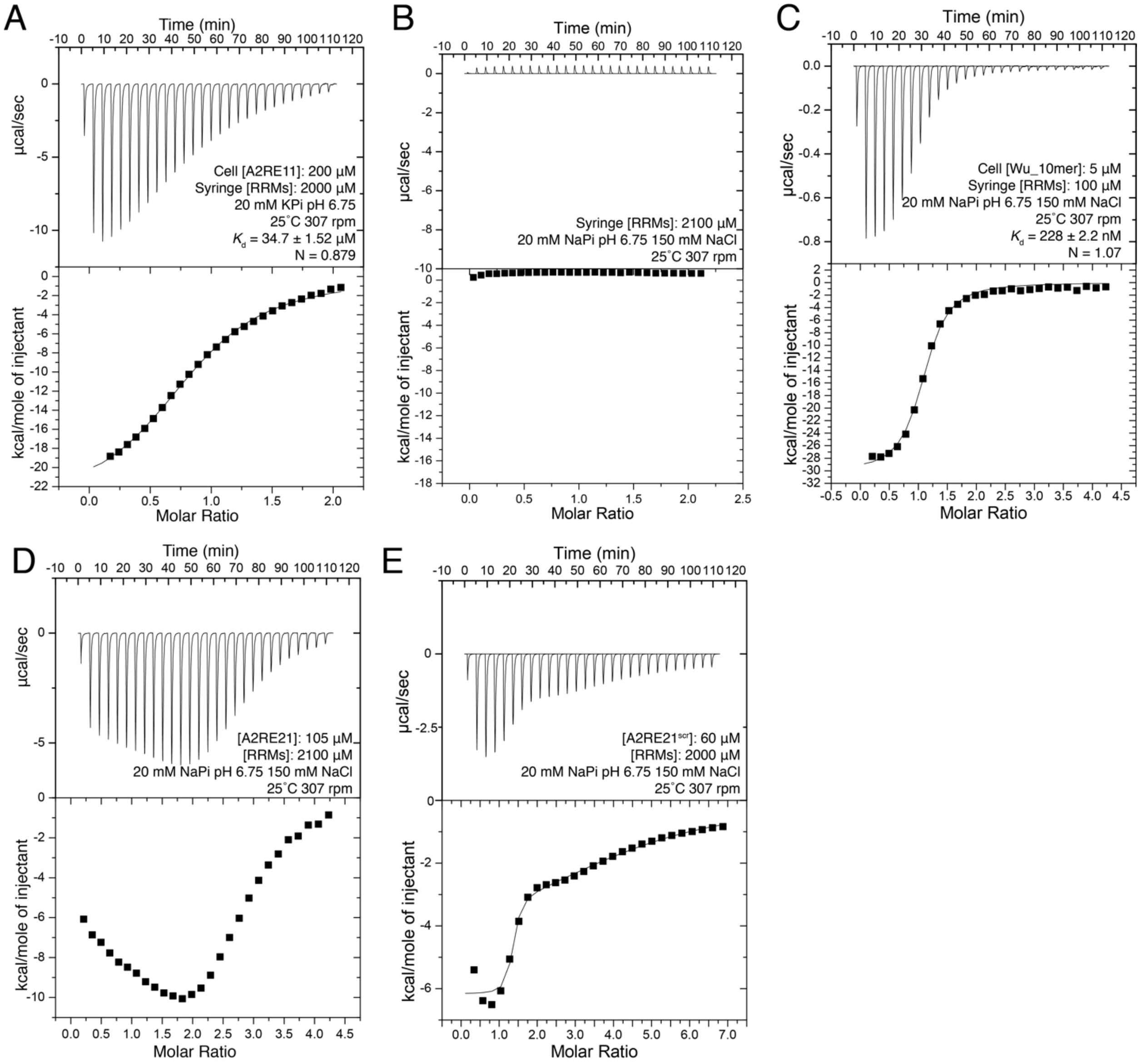

Figure S2: The RRMs of hnRNPA2 bind RNA weakly. Related to Figure 2.

A) ITC of the RRMs binding to rA2RE11 in a second buffer condition (lower salt concentration). Calculated binding affinity is about 35 µM and the interaction stoichiometry seems to be approximately 1 to 1, indicating that buffer and salt concentration does not drastically alter the binding of rA2RE11 to hnRNPA2 1-189.

B) ITC showing the heat of dilution of the RRMs. hnRNPA2 1-189 was titrated into buffer, demonstrating that there is no substantial baseline affecting our ITC runs with RNA.

C) ITC of hnRNPA2 1-189 binding to the Wu_10mer RNA (Wu et al 2018). This RNA shows tighter binding to hnRNPA2 1-189, with a K_d of approximately 230 nM, similar to the reported value.

D) ITC of the RRMs binding to rA2RE21 at a second concentration (higher A2RE21 cell concertation). Like at the lower rA2RE21 concentration (Figure 2B), the isotherm has a distinctive biphasic curve consistent with multiple binding sites and appears similar to that observed at lower cell concentration conditions.

E) ITC of the RRMs binding to $rA2RE21^{scr}$. hnRNPA2 1-189 does not substantially bind to the scrambled rA2RE21 sequence, demonstrating some sequence specificity of the interaction with rA2RE21, even though the affinity is weak.

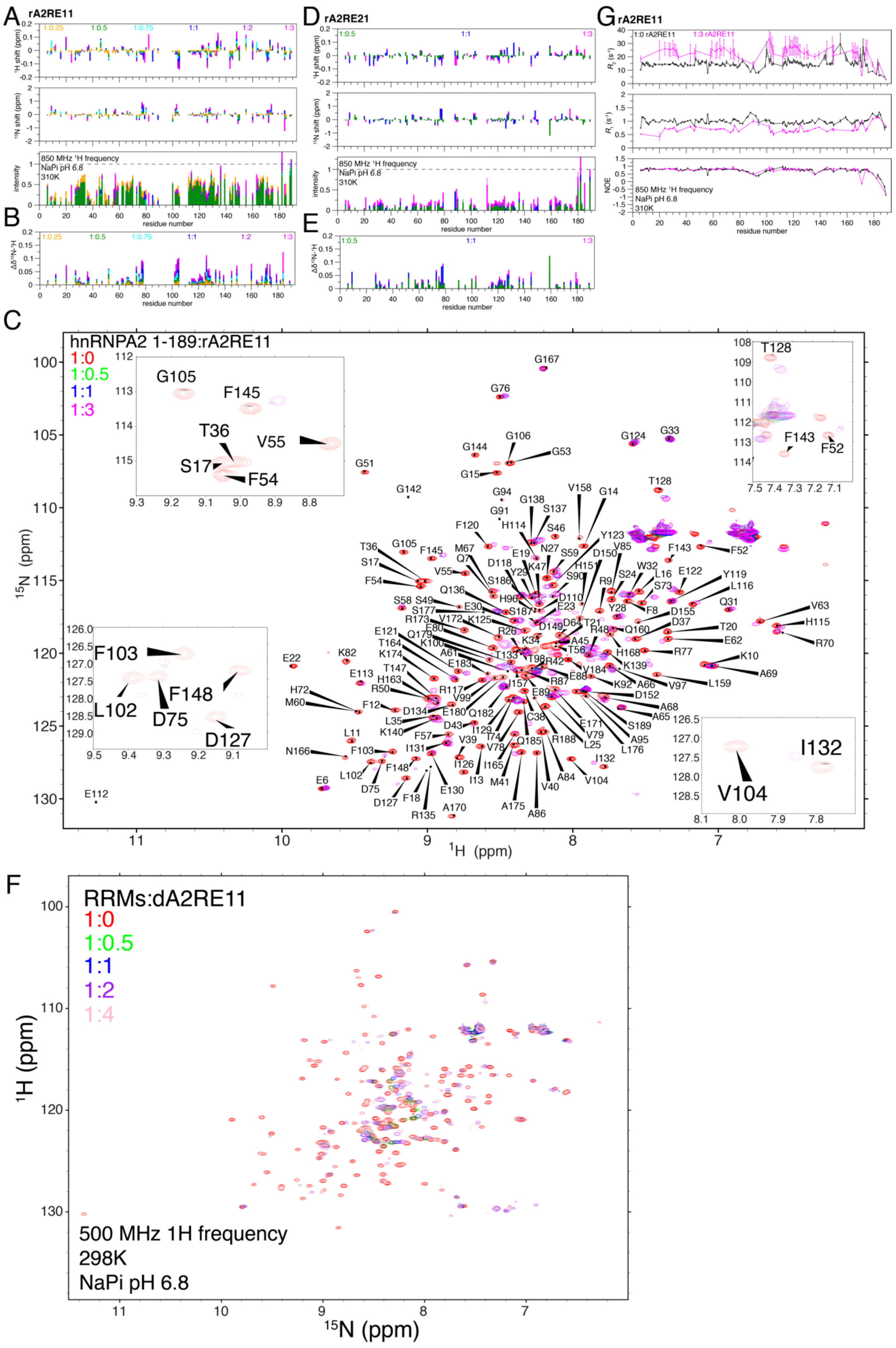

A rA2RE11
1:0.25 1:0.5 1:0.75 1:1 1:2 1:3
¹H shift (ppm)
¹⁵N shift (ppm)
intensity
850 MHz ¹H frequency
NaPi pH 6.8
310K
residue number
B
Δδ¹⁵N-¹H
D rA2RE21
1:0.5 1:1 1:3
E
G rA2RE11
1:0 rA2RE11
1:3 rA2RE11
R₂ (s⁻¹)
R₁ (s⁻¹)
NOE
C
hnRNPA2 1-189:rA2RE11
1:0
1:0.5
1:1
1:3
¹H (ppm)
¹⁵N (ppm)
F
RRMs:dA2RE11
1:0
1:0.5
1:1
1:2
1:4
500 MHz 1H frequency
298K
NaPi pH 6.8
¹H (ppm)
¹⁵N (ppm)

Figure S3: NMR data of hnRNPA2 RRMs binding RNA. Related to Figure 2.

A) Quantification of chemical shift deviations and peak intensity differences from the titration in Figure 2C. Most chemical shift deviations increase with increasing rA2RE11 concentration, indicative of fast exchange. Peak intensities are consistently weaker in the presence of RNA and some peaks reduce intensity and then increase intensity.

B) Average chemical shift deviations for (A).

C) ^{1}H-^{15}N HSQC of hnRNPA2 1-189 titrated with increasing concentrations of rA2RE21. Data is consistent with weak binding with most peaks being in intermediate-fast exchange. Insets show similar regions to those in Figure 2C.

D) Quantification of chemical shift deviations and peak intensity differences from a titration of the rA2RE21 with 1-189. Most chemical shifts increase with increasing rA2RE21 concentration, indicative of fast exchange. Peak intensities are consistently weaker in the presence of RNA and some peaks reduce intensity and then increase intensity, similar to what was observed with rA2RE11.

E) Average chemical shift deviations for (D).

F) ^{1}H-^{15}N HSQC titration of dA2RE11 binding to 1-189. Data is consistent with weaker binding than to rA2RE11, as many resonances broaden beyond detection and do not return at excess dA2RE11.

G) NMR spin relaxation parameters ^{15}N R_2, ^{15}N R_1, and hetNOE values for 1-189 at 200 μM without RNA (black) and with 3 times molar excess rA2RE11 RNA (magenta) indicate the complex with RNA is large and with slower tumbling. 850 MHz ^{1}H frequency, 310K.

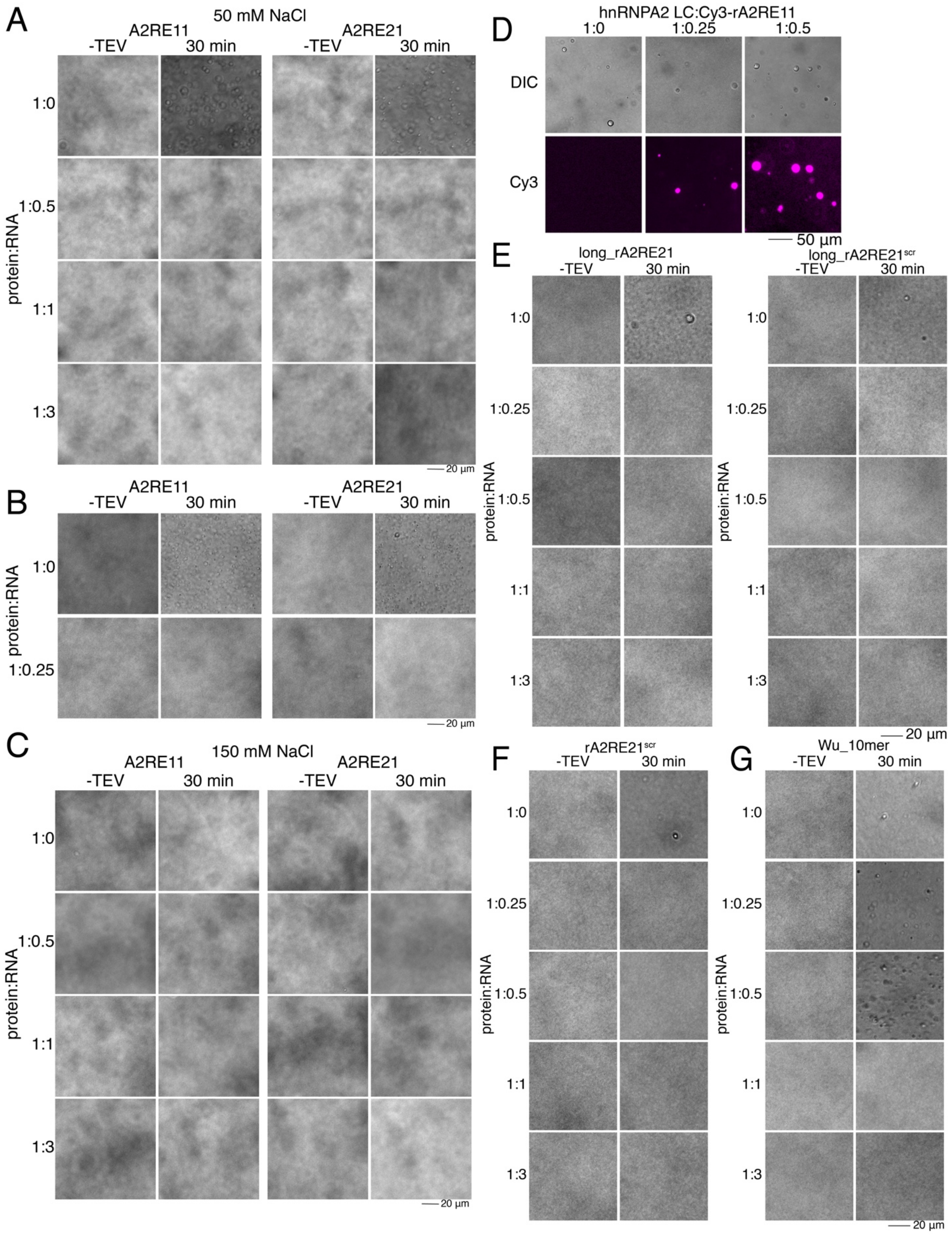

Figure S4: hnRNPA2 FL phase separates in lower salt conditions. Related to Figure 4.

A-B) hnRNPA2 FL WT phase separation is eliminated at all RNA concentrations tested at 50 mM NaCl. -TEV represents the negative control where the C-terminal maltose binding protein solubility tag is retained.

C) At 150 mM NaCl, hnRNPA2 FL WT does not undergo LLPS after cleavage of a C-terminal maltose binding protein solubility tag in the absence of RNA. When added, RNA is unable to induce phase separation.

D) In conditions where hnRNPA2 LC undergoes LLPS (20 μM protein, pH 5.5 MES 50 mM NaCl), Cy3-labeled rA2RE11 is able to partition into hnRNPA2 LC droplets.

E) Addition of longer (60 nucleotide) RNA oligomers containing the A2RE21 or the scrambled sequence, long_A2RE21 and long_A2RE21^{scr}, both eliminate LLPS of hnRNPA2 FL WT at all concentrations tested.

F) rA2RE21^{scr} eliminates LLPS of hnRNPA2 FL WT at all concentrations tested.

G) At protein:RNA ratios of 1:0.25 or 1:0.5, the Wu_10mer RNA does not eliminate LLPS of hnRNPA2 FL WT. However, at higher concentrations of RNA (where the sites on the RRMs for binding RNA are likely saturated), LLPS is eliminated.
